# Clinical radiographic outcomes and survivorship of medial pivot design total knee arthroplasty: a systematic review of the literature

**DOI:** 10.1007/s00402-021-04210-6

**Published:** 2021-10-11

**Authors:** Mattia Alessio-Mazzola, Antonio Clemente, Antonio Russo, Peter Mertens, Giorgio Burastero, Matteo Formica, Lamberto Felli

**Affiliations:** 1 Orthopedic Clinic, Ospedale Policlinico San Martino, Largo R. Benzi 10, 16132 Genoa, Italy; 2 Department of Surgical Sciences and Integrated Diagnostic (DISC), University of Genoa, Viale Benedetto XV 6, 16132 Genoa, Italy; 3 Orthopaedics and Traumatology Department, ZNA Middelheim, Antwerp, Belgium; 4 Centro di Chirurgia Protesica, Istituto Ortopedico Galeazzi IRCCS, Via Riccardo Galeazzi 4, 20161 Milan, Italy; 5 Istituto Ortopedico Galeazzi IRCCS Chirurgia Articolare Sostitutiva e Chirurgia Ortopedica, Via Riccardo Galeazzi 4, 20161 Milan, Italy

**Keywords:** Medial pivot knee, Medial pivot TKA, Medial congruent knee, Medial stabilized knee, TKA design, TKA kinematics

## Abstract

**Background:**

Total knee arthroplasty is a reliable procedure able to reduce pain and disability in patients suffering from osteoarthritis. However, a considerable percentage of patients still experiences unsatisfactory results. Medial pivot total knee arthroplasty has been introduced in the clinical practice to overcome problems related with classic design implants and better mimic native knee kinematics. The aim of this study was to analyze survivorship and clinical and radiographic outcomes of medial pivot implants.

**Methods:**

A systematic research was conducted in eight different databases. Thirty-four studies met the inclusion criteria and were included in the analysis. Data on objective and patients-reported outcomes, radiographic alignment, and survivorship were collected and analyzed. Revision rate was expressed as revision per 100 components years.

**Result:**

A total of 3377 procedures were included. Mean follow-up was 85.7 months (range, 12–182). The revision per 100 components years was 0.19, which corresponds to a revision rate of 1.9% after 10 years. Mean post-operative range of motion was 117.3 ± 0.4°. Mean clinical and functional Knee Society Score were, respectively, 85.9 ± 1.1 and 84.7 ± 3.5 at final follow-up. Post-operative femorotibial alignment was 177.1 ± 0.5°. Alfa and beta angles were 95.7 ± 0.1° and 89.2 ± 0.1°, respectively. Gamma and delta angles were 2.3 ± 0.6° and 86.7 ± 0.4°.

**Conclusion:**

Medial pivoting implants provided excellent survivorship and low revision rate, as well as good-to-excellent results in term of objective and patient-reported clinical outcomes, and reliable correction of radiographic parameters. More high-quality studies with long-term follow-up are needed to clarify the role of medial pivoting implants.

**Supplementary Information:**

The online version contains supplementary material available at 10.1007/s00402-021-04210-6.

## Introduction

Total knee arthroplasty (TKA) represents a safe and reliable procedure to reduce pain and functional limitation caused by end-stage osteoarthritis (OA) [1]. The incidence of primary TKA is 450/100,000 and annual rates of surgical procedures are widely increasing worldwide [2–4].

Despite the continuous studies to develop new prosthetic designs with advanced kinematic concepts, unsatisfactory results are still reported in 20% of patients undergone TKA [5]. The importance of a reliable prosthetic design together with surgical and medical strategies has been emphasized to improve the functional outcome and achieve better clinical results in TKA [6].

Native kinematic of the medial compartment of the knee is a “ball-and-socket” mechanism, with medial femoral condyle constrained in a pivot motion, while the lateral femur is free to translate posteriorly through a complete arc of flexion [7].

The medial pivot design was introduced in 1994 miming the physiological knee kinematic, to ensure greater efficacy of extensor mechanism in full range of motion (ROM) [8, 9]. This philosophy is based on femoral component with single- or multi-radius curve and a tibial insert with a highly congruent medial compartment and flat lateral compartment. The anteroposterior stability is ensured by a raised anterior lip of polyethylene with minimum risk of condylar lift off [9, 10].

Several studies showed promising mid-term results of medial pivot TKA [11], but the long-term survivorship and clinical outcome have not been extensively investigated with high level of evidence.

The purpose of this systematic review is to analyze survivorship and clinical and radiographic outcomes of medial pivot design TKA.

## Material and methods

### Literature search and inclusion criteria

**Fig. 1:**
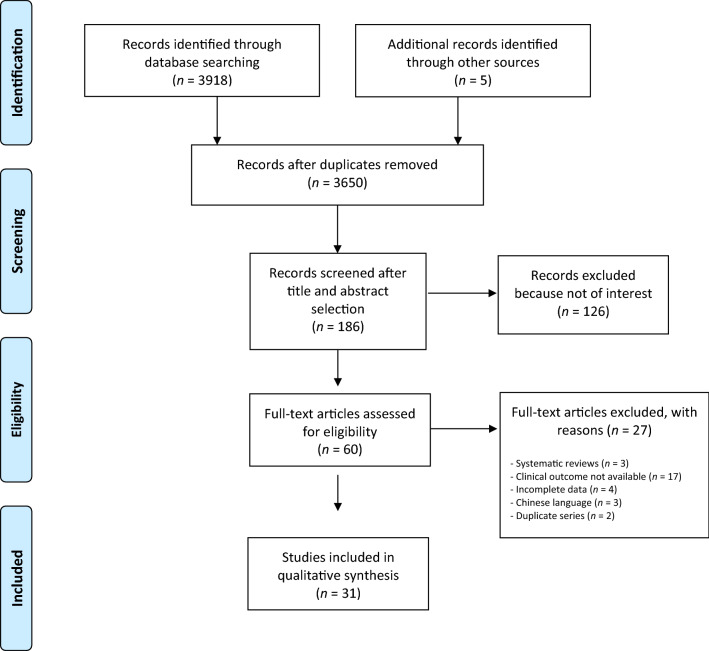
PRISMA flow diagram

A systematic review of the literature has been performed, following *Cochrane Handbook of Systematic Reviews of Interventions* [12] and Preferred Reporting Items for a Systematic Reviews and Meta-Analyses (PRISMA) [13] for study selection (Fig. 1).

A systematic search from January 1st, 1990, until October 1st, 2020, was performed in the following databases: the Cochrane Central Register of Controlled Trials (CENTRAL), MEDLINE/PubMed, Embase, Scopus, the Science Citation Index Expanded from Web of Science, ScienceDirect, CINAHL, and LILACS. The research was conducted using the following keywords alone and in all the various combinations: “Medial pivot knee”, “TKA design”, “medial congruent knee”, and “medial stabilized knee”.

Clinical studies reporting objective and patient-reported outcome of medial pivot design TKA were considered eligible for inclusion. There was no quality restriction for study inclusion. Case report, technical notes, editorial commentaries, ex vivo, biomechanical, pre-clinical, and clinical studies without adequate quantitative or qualitative data were excluded. Studies that did not report clear clinical-functional data or survivorship of primary medial pivot TKA were excluded from this research.

Two reviewers independently screened each title and abstract collected from the primary electronic search. In case of relevant title and abstract, the full-text version was obtained.

All references of each study were screened to find any additional relevant paper potentially missed with the first review process. The two reviewers independently followed the same checklist to screen all studies and evaluate the eligibility criteria. Disagreements were resolved through discussion with third reviewer.

The primary endpoints of this analysis were the survivorship and clinical outcome of medial pivot design TKA and revision rate. Secondary endpoints were radiological alignment and complications.

### Appraisal of studies’ quality and risk of bias

The level of evidence of included studies were evaluated through the adjusted Oxford Centre For Evidence-Based Medicine 2011 Levels of Evidence [14]. The quality of the studies was defined using the Grading of Recommendations Assessment, Development, and Evaluation (GRADE) system [15], rating quality of evidence in systematic reviews. After the evidence is collected and summarized, the GRADE system provides explicit criteria for rating the quality of evidence that include study design, risk of bias, imprecision, inconsistency, indirectness, and magnitude of effect.

The risk of bias was assessed with “Risk Of Bias” (Rob 2.0) for Randomized Trial (RCT) and “Risk of bias in non-randomized studies” (ROBINS-I) [16] to evaluate non-RCT studies.

Relevant conflict of interest having potential impact on study design and results were considered and reported.

### Data extraction and analysis

A stepwise analysis on study design, aim of study, level of evidence, journal, number of procedures included in the study, prosthetic implant used, indication to TKA, mean age, body mass index (BMI), follow-up, and patellar resurfacing. Disparities in data extraction were discussed and resolved by consensus meeting between the authors. When studies reported mixed cohorts of patients, data extraction was selectively focused on procedures involving medial pivot implants.

Radiological data reported as pre- and post-operative femoral tibial angle (FTA), implant alignment with alpha angle (*α*), femoral beta angle (*β*), sagittal femoral gamma angle (*γ*), and sagittal tibial delta angle (*δ*) were collected. The femoral and tibial radiolucent lines (RLL) were also noted, and divided into major and progressive and minor and non-progressive, according to the knee society total knee arthroplasty roentgenographic evaluation and scoring system [17]. Continuous variables were expressed as weighted means and weighted standard deviation. Mean survivorship, clinical-functional outcomes, and radiographic angles were calculated as weighted means. All studies were assessed for revision for any reason. To evaluate survival rate of implants included in studies with different follow-up times, revisions per 100 components years (CY), which is a well-established method in orthopedic literature [18], were calculated. Studies missing data on the number of revisions performed were excluded from this analysis.

## Results

The database research identified a total of 3655 studies. After initial screening, 191 studies were retrieved for full assessment. A total of 34 studies were included in the systematic review. Details are reported within Table 1. Two studies were level of evidence I [10, 19], 8 studies level II [9, 20–26], and 24 level IV [8, 11, 27–48].

**Table 1:** General characteristics of included studies

Main author	Year	Study design	Patients (*n*)	TKA procedure (*n*)	Mean age (YEARS)	Follow-up (months)	Survivorship (%)	Risk of bias (robins-I/Rob2)	Level of evidence (CEBM)
*Mannan*	2009	Prospective case series	189	228	–	72	94.5	Serious	IV
*Fan*	2010	Retrospective case series	56	58	65.1	64.7	–	Serious	IV
*Hossain*	2011	RCT	40	40	72.5	24	100	Low	IIb
*Vecchini*	2012	Prospective case series	160	172	71	84	–	Moderate	IV
*Ishida*	2012	RCT	20	20	71	57	–	Some concerns	Ib
*Brinkman*	2013	Prospective case series	47	50	69	119	98	Serious	IV
*Youm*	2014	Prospective case series	80	120	66.4	64.7	99.9	Serious	IV
*Chinzei*	2014	Retrospective case series	76	85	70.2	93.1	–	Serious	IV
*Schmidt*	2014	Prospective case series	320	365	66.5	54	96.6	Serious	IV
*Bae*	2015	Prospective case–control	125	150	66.7	63	98.6	Serious	IV
*Katchky*	2016	Prospective case series	81	87	68	60	98	Serious	IV
*Nakamura*	2016	Retrospective case series	51	70	82	142	–	Serious	IV
*Choi*	2016	Retrospective case–control	28	49	66.7	64	–	Serious	IV
*Karachalios*	2016	Retrospective case series	195	251	71	161	96.4	Serious	IV
*Macheras*	2017	Retrospective case series	325	347	78	182	98.8	Serious	IV
*Dehl*	2017	Retrospective case series	48	50	66.5	114	93	Serious	IV
*Kim*	2017	RCT	182	182	65.6	144	99	Some concerns	IIb
*Benjamin*	2018	RCT	45	45	62.4	12	100	Low	Ib
*Nakamura*	2018	Retrospective case–control	45	45	74.3	24	100	Serious	IV
*Samy*	2018	Prospective case series	76	76	64.4	12	98.5	Moderate	IV
*Kohei*	2018	RCT	33	33	73.8	24	100	Some concerns	IIb
*Sabatini*	2018	Retrospective case series	10	10	–	12	–	High	IV
*Karachalios*	2018	Prospective case–control	54* 54**	54* 54**	63.2* 63.8**	79* 79*	100* 100**	Serious	IV
*Cacciola*	2019	Retrospective case series	297	315	74	66,4	98.3	Moderate	IV
*Indelli*	2019	RCT	50	50	67.3	24	100	Some concerns	IIb
*Gill*	2019	RCT	35	35	68.8	24	-	Some concerns	IIb
*French*	2019	RCT	46	46	69	13.1	100	Some concerns	IIb
*Yuan*	2019	Retrospective case–control	49	49	69.43	60	100	Moderate	IV
*Indelli*	2020	Retrospective case–control	50^†^ 50^††^	50^†^ 50^††^	68.5^†^ 67.3^††^	24^†^ 24^††^	100^†^ 98^††^	Serious	IV
*Lee*	2020	RCT	23	23	70	12	–	Some concerns	IIb
*Jones*	2020	Prospective case–control	30	30	69.6	13.2	–	Serious	IV
*Risitano*	2020	Prospective case–control	15	15	73.5	12	100	Serious	IV
*Edelstein*	2020	RCT	25	25	67	24	–	Some concerns	IIb
*Jeremic*	2020	Prospective case–control	24^a^ 24^b^	24^a^ 24^b^	70.7^a^ 72.5^b^	12^a^ 12^b^	100^a^ 100^b^	Serious	IV

*RCT* Randomized Controlled Studies, *TKA* Total Knee Arthroplasty

*Cementless components cohort, **cemented component cohort, ^†^J curve design cohort, ^††^single radius Design cohort, ^a^kinematically aligned, ^b^mechanically aligned

The overall quality of the included study was very low according to GRADE system. Twenty-two studies were rated as very low quality [8, 11, 27, 28, 30–45, 47, 48], 6 low quality [22–24, 26, 29, 46], 4 moderate [9, 10, 21, 25], and 2 high quality [19, 20] (Table 1). The risk of bias was considered high in 1 research [11], serious in 19 [8, 27, 30–36, 38–45, 47, 48], and moderate in 4 [28, 29, 37, 46]; 9 [9, 19–26] had some concerns and one RCT [10] had low risk of bias.

A total of 3058 patients (3377 medial pivot design TKA) were finally included in this systematic review.

The mean age at surgery was 69.9 ± 4.4 years and the mean BMI was 29.9 ± 1.0 kg/m^2^. The mean follow-up was 85.8 months (range, 12–182). Patellar replacement was described in 23 studies [9–11, 20, 21, 23, 24, 26, 28, 30, 33, 35–40, 42–46], and it was performed in 880 (44.0%) cases on a total of 2000 procedures.

### Survivorship and revision rate

The revisions per 100 CY were 0.19, which correspond to a revision rate of 1.9% after 10 years (Table 2). The causes of revision were: 16 cases of periprosthetic joint infection (PJI), 9 cases of aseptic loosening, 8 cases of periprosthetic fractures, 6 cases of persistent pain, 3 component failures (at least one TKA component), and 2 instabilities. Seven of the included studies did not report appropriate data on revisions rate, and then, these studies were not included in the calculation of overall revisions per 100 CY.

**Table 2:** Revisions per 100 observed components years of the included studies

Main author	Follow-up (year)	Number of procedures	CY	Number of revisions	100 Revision/CY
*Mannan*	6	228	1368	11	0.80
*Fan*	5.4	58	313.2	0	0
*Hossain*	2	40	80	0	0
*Vecchini*	7	172	1204	2	0.17
*Ishida*	4.8	20	96	0	0
*Brinkman*	9.9	50	495	1	0.20
*Youm*	5.4	120	648	1	0.15
*Chinzei*	7.8	85	663	1	0.15
*Schmidt*	4.5	365	1642.5	7	0.43
*Bae*	5.3	150	795	2	0.25
*Katchky*	5	87	435	2	0.46
*Nakamura*	11.8	70	826	1	0.12
*Choi*	5.3	49	259.7	NA	NA
*Karachalios*	13.4	251	3363.4	6	0.18
*Macheras*	15.2	347	5274.4	4	0.08
*Dehl*	9.5	50	475	3	0.63
*Kim*	12	182	2184	1	0.05
*Benjamin*	1	45	45	NA	NA
*Nakamura*	2	45	90	0	0
*Samy*	1	76	76	0	0
*Kohei*	2	33	66	NA	NA
*Sabatini*	1	10	10	0	0
*Karachalios*	6.6	108	712.8	0	0
*Cacciola*	5.5	315	1732.5	2	0.12
*Indelli*	2	50	100	0	0
*Gill*	2	35	70	NA	NA
*French*	1.1	46	50.6	0	0
*Yuan*	5	49	245	0	0
*Indelli*	2	100	200	0	0
*Lee*	1	23	23	NA	NA
*Jones*	1.1	30	33	NA	NA
*Risitano*	1	15	15	0	0
*Edelstein*	2	25	50	NA	NA
*Jeremic*	1	48	48	0	0

*CY* components years, *NA* not available, *y* years

### Functional outcomes

The pre-operative mean ROM was 103.4 ± 1.5 (CI 95% 103.3–103.5°). Mean pre-operative KSS and KSS for function were 38.7 ± 1.7 (CI 95% 38.6–38.9) points and 45.8 ± 4.4 (CI 95% 45.7–45.9) points, respectively, and mean pre-operative OKS was 38.6 ± 8.6 (CI 95% 38.2–39.0) points. Considering post-operative results, mean ROM was 117.3 ± 0.4° (CI 95% 117.29–117.31), clinical and functional KSS were 85.9 ± 1.1 (CI 95% 85.88–85.92), and 84.7 ± 3.6 (CI 95% 84.6–84.8). WOMAC and KOOS values were 39.1 ± 7.6 (CI 95% 38.8–39.4) and 84.9 ± 2.2 (CI 95% 84.6–85.2), OKS was 28.2 ± 5.7 (CI 95% 27.9–28.5), and FJS mean value was 68.5 ± 1.0 (CI 95% 68.4–68.6).

Mean ROM of RCTs improved from 112.2 ± 7.2° (CI 95%111.5–112.9) preoperatively to 115.9 ± 1.6° (CI 95% 115.7–116.1) at final follow-up, while clinical and functional KSS varied from 34.0 ± 5.1 (CI 95% 33.3–34.7) and 44.7 ± 0.2 (CI 95% 44.6–44.8) before surgery to 87.7 ± 1.0 (CI 95% 87.6–87.8) and 78.1 ± 2.6 (CI 95% 77.8–78.4). OKS improved from 25.9 ± 0.9 (CI 95% 25.7–26.1) to 35.9 ± 1.5 (CI 95% 35.7–36.1).

Detailed functional outcomes are reported within Table 3.

**Table 3:** Clinical Outcomes of included studies

	Preoperative							Post-operative								Mean follow-up (m)
Main author	ROM (°) (± sd)	KSS C (± sd) F (± sd)		WOMAC (± sd)	HSS (± sd)	KOOS (± sd)	OKS (± sd)	ROM (°) (± sd)	KSS C (± sd) F (± sd)		WOMAC	HSS	KOOS	FJS	OKS	
											(± sd)	(± sd)	(± sd)	(± sd)	(± sd)	
RCTs																
* Hossain*	97.3 (15)	43 (13)	44.6 (15)	56 (17.3)	/	/	41.6 (7.5)	114 (12.8)	76.3 (15.5)	71.4 (15.8)	27.1 (9.1)	/	/		26.2	24
* Ishida*	110	34	40	/	/	/	/	110	89	65	/	/	/	/	/	57
* Kim*	124	29	44.8	61	/	/	/	117	90	80	25	/	/	/	/	144
* Benjamin*	/		54.2	/	/	/	29.7	/	/	85.1	/	/	/	/	39.6	12
*Kohei*	98.1 (21.9)	39 (19.0)	44 (24.5)	/	/	/	/	108.7 (15.8)	85.1 (10.0)	74 (19.4)	/	/	/	/	/	24
*Indelli*	108	63.7		/	/	/	19	123	165.7		/		/	/	41	24
*Gill*	/	/	/	/	/	/	/	119 (3.1)	/	89.2 (1.7)	/	/	/	60.0 (16.7)	/	24
*French*	102 (8.9)	/	/	50.5 (16.3)	/	35.4 (15.6)	20 (8.7)	115 (10.0)	/	/	8.6 (9.5)	/	84.6 (13.4)	79.9 (20.4)	42 (5.0)	13.1
*Lee*	97 (15)	51 (19)	49 (12)	49 (19)	/	/	/	108 (12)	91 (11)	58 (21)	19 (14)	/	/	75 (24)	/	12
*Edelstein*	/	/		/	/		16.3 (7.6)	/	88.1 (9.5)	81.4 (17.9)	/	/		57.1 (37.6	22.8 (9.6)	24
Level IV evidence																
*Mannan*	/	47.6	45.1	/	/	/	/	/	72.2	93.1	/	/	/	/	/	72
*Fan*	103 (2.0)	30.5 (2.3)	36.7	/	/	/	/	115 (1.8)	91.1 (1.3)	82.3	/	/	/	/	/	64.7
*Vecchini*	97.7	28.3	49.1		/	/		112.5	73.2	79.9		/	/			84
*Brinkman*		33.5	50	34	/	/	/	110	84.0	80	22	/	/	/		119
*Youm*	107.5	46.6	38.6	54.8	/	/	/	119	87.4	82.0	18.3	/	/	/	/	64.7
*Chinzei*	94.2	36.2	31.4	/	/	/	/	110.6	92.1	73.4	/	/	/	/	/	93.1
*Schmidt*	110	/	67.1	/	/	/	/	115	/	95.5	/	/	/	/	/	54
*Bae*	114 (14.3)	59.9 (7.5)	53.3 (7.1)	32.9 (4.8)	/	/	/	124 (14.3)	90.0 (6.6)	85.6 (8.5)	14.3 (5.7)	/	/	/	/	63
*Katchky*	/	/	/	44 (17)	/	51 (13.1)	22 (7.4)	124	/	/	6.5 (9.1)	/	88.6 (13.1)	75.3 (28.3)	44 (3.9)	60
*Nakamura*	104 (23)	14 (13)	47 (13)		/	/		116 (23)	89 (11)	68 (21)		/	/			24
*Choi*	114 (15.7)	40.6 (9)	51.9 (12.5)	59.1 (11)	/	/	/	121 (11.7)	89.4 (7.6)	88.8 (10.1)		/	/	/	/	64
*Karachalios*	101	31	42.9	30.8	/	/	44.4	117	89.2	78.4	69.2	/	/	/	25,1	161
*Macheras*	85	32.5 (12.2)	42.7 (12.9)	30.7 (9.8)	/	/	44.5 (5.0)	120	92 (7.9)	82 (16.2)	79.3 (17.3)	/	/		21.9 (9.1)	182
*Dehl*	98.5	60.68	48.46	/	/	/	/	110 (3)	90.34	104	/	/	/	/	/	114
*Nakamura*	104 (23)	55 (14.3)	33.3 (21.1)	/	/	/	/	119.3	/	92.2	/	/	/	/	/	24
*Samy*	120 (17.8)	/	/	/	/	/	/	121.7 (21.5)	/	/	/	/	/	60.5 (31.4)	/	12
*Sabatini*	/	64.4		/	/	/	19.5	124	167.5		/		/	/	41.2	12
*Karachalios*	101*	35.6*	46.4*	31.8*	/	/	44.3*	116*	98.1*	97*	69.2*	/	/	/	22*	161
	108**	32**	46**	34**	/	/	43.8**	118**	95**	95.1**	70**	/	/	/	23,3**	161
*Cacciola*	98	39	33.4	48.9	/	/	46	118	81.7	90.6	12.2	/	/	67.3	24	66.4
*Yuan*				100 (17.4)	47.0 (12.5)	/	/				26	93				60
*Indelli*	108^†^	63^†^	43^†^	/	/	/	19^†^	123^†^	87^†^	78^†^	/	/	/	/	41^†^	24
	110^††^	64^††^	45^††^	/	/	/	20^††^	116^††^	84^††^	75^††^	/	/	/	/	38^††^	24
*Jones*	/	/	/	/	/	/	/	/	/	/	5.0 (6.1)	/	91.1 (9.2)	84 (18.1)	43.6 (3.4)	13.2
*Risitano*	/	41.0 (4.0)	51.0 (6.2)	/	/	/	20.2 (5.5)	123 (5.3)	89.1 (6.3)	81.8 (8.4)	/	/		79.3 (3.3)	41.3 (2.1)	12
*Jeremic*	/	35.6 (24.7)^a^	29.4 (20.7)^a^	/	/	29.3^a^	/	/	94^a^	67^a^	/	/	81.7^a^	77^a^		12
	/	29.4 (12)^b^	27.2 (10.0)^b^	/	/	27.6^b^	/	/	75^b^	60^b^	/	/	67.2^b^	51^b^		12

*C* Clinical, *F* Functional, *FJS* Forgotten Joint Score, *FU* follow-up, *HSS* Hospital for Special Surgery, *KOOS* Knee Osteoarthritic Outcome Score, *KSS* Knee Society Score, *OKS* Oxford Knee Score, *ROM* Range of motion, *sd* standard deviation, *WOMAC* Western Ontario and McMaster University Osteoarthritic Index

*Cementless components cohort, **cemented component cohort, ^†^J curve design cohort, ^††^single radius design cohort, ^a^kinematically aligned, ^b^mechanically aligned

### Radiographic outcomes

Eight studies [19, 23, 27, 35, 39, 41, 43, 48] reported a pre-operative varus deformity (699 TKA [47.4%], mean FTA value: 186.9 ± 0.4° [CI 95% 186.8–187.0]), and other 6 [9, 21, 29, 36, 38, 44] reported a valgus FTA (774 TKA [52.6%], mean FTA 174.6 ± 0.3° [CI 95% 174.5–174.7]). The overall pre-operative FTA was 180.5 ± 0.8°. Mean post-operative FTA after 6.9 years was 177.1 ± 0.1° (CI 95% 177.0–177.2). Post-operative alfa and beta knee angles mean values after 8,4 years were 95.6 ± 0.4° (CI 95% 95.5–95.7) and 89.1 ± 0.2° (CI 95% 89.0–89.2), respectively, while the gamma and delta angles and their mean values were 2.3 ± 0.7° (CI 95% 2.2–2.4) after 8.4 years and 86.7 ± 0.4° (CI 95% 86.6–86.8) after 8.7 years.

Minor (< 2 mm) and non-progressive femoral radiolucent lines were found in 146 (7.5%) knees on the femoral side and in 175 (9.0%) cases on the tibial side. Major or progressive femoral RLL were reported in 11 (0.5%) cases. Two studies [8, 35] reported 12 (0.6%) RLL minor case without mentioning the exact localization.

Post-operative outliers were 116 (24.3%) cases (mechanical axis alignment ± 3 degrees). Regarding RCTs, the FTA varied from 188.1 ± 5.7° (CI 95% 187.4–188.8) after the procedure to 175.9 ± 2.3° (CI 95% 175.2–176.6) after 9.1 years.

Post-operative alfa and beta angles were 96.6 ± 2.2° (CI 95% 96.3–96.9) and 88.6 ± 0.1° (CI 95% 88.5–88.7), while gamma and delta values were 2.8 ± 0.4° (CI 95% 2.7–2.9) and 86.5 ± 0.5° (CI 95% 86.4–86.6). In RCT, studies were not reported any case of RLL or AL. Details of radiological measurements are reported within Table 4.

**Table 4:** Radiographic outcomes of included studies

Main author	Preoperative	Post-operative				
	FTA (°), (± sd)	Alfa (°) (± sd)	Beta (°) (± sd)	Gamma (°) (± sd)	Delta (°) (± sd)	FTA (°) (± sd)
RCTs						
*Kohei*	Valgus 9 (4.1)	89.4 (1.7)	89.1 (2.1)	4.6 (2.8)	86.3 (3.1)	Varus 1 (2.3)
*Hossain*	Valgus 4.0 (4.3)	95.6 (3.9)	88.4 (1.9)	2.4 (2.7)	88.7 (4.3)	/
*Ishida*	Varus 12	/	/	/		Varus 1
*Kim*	Varus 10.8	98.1	88.6	2.5	86.1	Valgus 5.6
*Nakamura*	181.3 (5.2)	100	88.1	6.2	87.8	174.2
*Cacciola*	4.5 valgus	96.8	88.4	1.6	88.7	Varus 2.8
*Sabatini*	/	/	/	/	/	Valgus 4
*Indelli*	/	/	/	/	84	Valgus 4.2
	/	/	/	/	87	Valgus 4.2
*Katchky*	/	/	/	/	/	Varus 2°
*Choi*	Varus 5.9 (4.0)	97.1 (3.4)	89.9 (1.5)	4.5 (3.6)	85 (1.1)	Valgus 5.6
Level IV evidence						
*Mannan*	Valgus 6.4	96.6	89	3.4	88.3	Valgus 5.6
*Vecchini*	/	88.2	94	/	/	/
*Dehl*	175	96.8	87.6	5.8	86	179
*Youm*	Varus 4.6 (4.5)	96.2 (2.1)	89.1 (1.7)	2.5 (1.5)	84.4 (2.7)	Valgus 5.8 (2.4)
*Chinzei*	10.7	/	/	/	/	1.4
*Bae*	Varus 4.1	95.3	90.1	3.0	84.8	Valgus 5.6
*Karachalios*	Valgus 5	97	88.5	1	85	Valgus 4.7
	Valgus 5.2	97	89	1	85	Valgus 4.8
*Macheras*	/	95	88.5	1	87.5	/
*Karachalios*	/	97	88.5	1	85.5	Valgus 4.5
*Risitano*	/	/	/	/	/	Varus 1.8
*Jeremic*	Varus 4.9^a^ Varus 5.2^b^	91.5^a^ 90.1^b^	88.4^a^ 89.0^b^	/	/	Varus 0.2^a^ Varus 0.15^b^
				/	/	

*FTA* Femoral–tibial angle, *sd* standard deviation

^a^kinematically aligned, ^b^mechanically aligned

### Complications

The main complications were 30 (1.0%) cases of stiffness, 25 (0.9%) cases of deep vein thrombosis, 17 (0.6%) PJIs, 17 (0.6%) superficial wound infections, 16 (0.6%) cases of persistent pain, 16 (0.6%) retarded wound healings, and 11 (0.4%) periprosthetic fractures.

Other reported complications were 7 (0.2%) cases of pulmonary thromboembolism, 6 (0.2%) AL, 6 (0.2%) peroneal neurapraxias, 4 (0.1%) cases of persistent knee swelling, 3 (0.1%) cases of patellar fractures, 3 (0.1%) cases of knee instability, 1 regional pain syndrome, and 1 patellar tendon rupture. In RCT studies, 14 (3%) cases of stiffness and 4 PJI were reported. Of these, three patients needed reoperation.

## Discussion

The aim of this systematic review was to summarize the literature evidence on survivorship and clinical–radiological outcomes of the medial pivoting design TKA. To the best of our knowledge, this is the first systematic review of the literature with a detailed ROM report, patient-reported and objective outcome measures, radiological outcomes, and complications of patients who underwent medial pivot TKA.

Although knee replacement is one of the most performed surgical procedures worldwide, some concerns are related to the relatively high percentage of unsatisfactory outcomes [49, 50]. Native knee kinematics is complex and consists of a constrained pivoting medial compartment and a lateral femoral condyle which can slide posteriorly at high grades of flexion [51, 52]. Medial pivoting designs have been proposed to mimic native knee kinematic and potentially improve clinical outcomes of classic PS TKA designs.

**Fig. 2:**
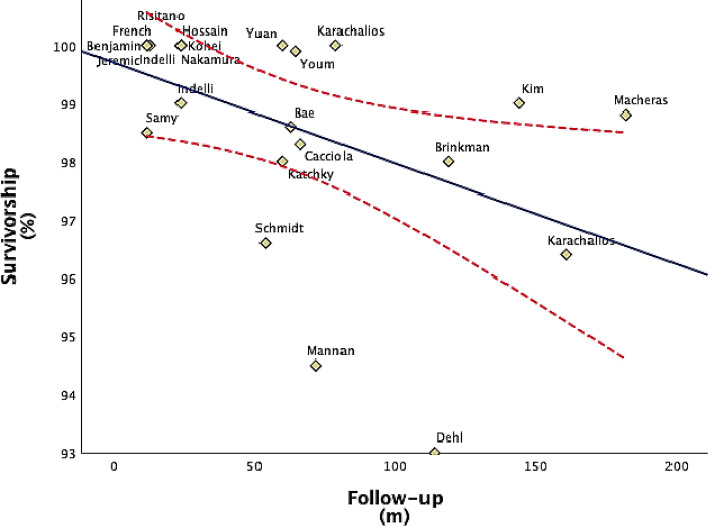
Scatterplot representing the relationship between survivorship and follow-up in each study included in the review. *Solid line*, linear median value of survivorship at different follow-up times. *Dotted line,* 95% CI of median survivorship calculated with the Wilcoxon *t* test

The most important finding of this research is the excellent overall survivorship of medial pivoting design TKA. In fact, the revisions per 100 CY were 0.19, corresponding to a revision rate of 1.9% after 10 years (Fig. 2). Only 51 revision TKA procedures were reported in the literature included in the current review. However, despite the overall excellent survivorship of these implants, survival analysis showed some outliers, as represented in Fig. 2 [8, 36, 38]. In particular, the mean survivorship reported in the retrospective study by Dehl et al. [38] was 93.0% at the final 9.5 year follow-up which is lower than the median value of the overall population studied. However, it should be considered that the small sample size of this study could have overestimated the revision rate, which main causes were not related to the implant design, such as arthrofibrosis and infections. Moreover, it should be highlighted that median values are not significantly affected by the presence of outliers. Some values points reported in the scatterplot (Fig. 2) are outside the 95% CI, showing the quite large dispersion of values around the line representing the projected median; then, any further conclusion based on the data presented in this review should be weighted considering this evidence. Nevertheless, it must be reminded that the 95% CI is a tool to assess the method to esteem values; then, real values should not be expected to be included into the interval.

Furthermore, medial pivot TKA showed good-to-excellent results in term of objective and patient-reported outcomes measures (Table 3). The RCTs included in this review demonstrated good-to-excellent clinical results of the medial pivot TKA, with strong improvement of KSS (from 34.0 to 87.7) and slight increase of ROM (from 112° to 115°). However, we point out that on a total of 9 RCTs included, only three studies demonstrated that the medial pivoting TKA had a better clinical results when compared to other knee implants (posterior stabilized and cruciate retaining) [9, 24, 25]. French et al. [25] reported significantly better FJS and quality of life subscale of the KOOS and KOOS-12 in a subgroup of patients who had undergone medial pivot TKA compared to cruciate retaining TKA. However, other patient-reported outcomes and ROM were comparable between the groups. Gill et al. [24] found better results in the group treated with medial pivot implants demonstrating better KSS, ROM, and FJS. On the contrary, Kim et al. [23] observed higher complication rates and worse knee scores, ROM, and patient satisfaction in the medial pivot design group than in the cruciate retaining group. Jeremic et al. [48] reported higher 1 year performance of kinematically medial pivot TKA compared to mechanically aligned ones.

A potential conflict of interest was disclosed in ten studies [9, 10, 21, 25, 28, 38, 42, 44, 45, 48] where authors reported receipt of payment, either direct or indirect, institutional support, and association with a biomedical field entity related to the medial pivot TKA, raising some concerns and precluding the reliable interpretation of clinical results.

Hossain et al. [9] found better ROM in the medial pivot TKA than in the posterior stabilized design (114.9° vs 100.1°). Moreover, physical component scores of SF-36 and Total Knee Function Questionnaire were better in the medial conforming ball-and-socket group. However, no differences were found in the American Knee Society, WOMAC, and Oxford Knee scores. Benjamin et al. [10] performed a gait analysis comparing the medial pivot TKA with the single radius PS implant. They found no significant differences in cadence, walking speed, stride length and stance time, peak stride, mid-support, and push-off forces.

This study has several limitations. First, there is a low level of evidence among included studies, since 22 papers were classified as level of evidence IV. Moreover, only one research was considered at low risk of bias precluding strong conclusions on the results of the included studies. No quality restriction was applied to obtain the largest population of medial pivot TKA. There are possible selection biases deriving from different diagnosis and high heterogeneity in TKA indications that include population (i.e., comorbidities, age, and pre-operative level of activity).

Heterogeneous RCTs studies have been included where the medial pivoting design was compared to the conventional posterior stabilized and cruciate retaining TKA or compared to other models of the medial pivot TKA. All reported outcome measures were highly heterogeneous resulting in a difficult systematic analysis. To reduce bias, the largest number of procedures available in the literature were included and variables gathered from RCTs were analyzed separately.

Conflict of interests were disclosed in several included studies, and this aspect can overestimate the medial pivot TKA outcomes. Only six studies [23, 33, 34, 38, 42, 45] reported survivorship and complications over 10 years raising some concerns of long-term failure risk of the medial pivot TKA.

## Conclusions

Medial pivoting design TKAs provided high survivorship of implants, with a revision rate of 1.9% after 10 years. Moreover, good-to-excellent results were obtained in term of objective and patient-reported outcomes measures. Radiological evaluation of studies showed excellent post-operative correction of axial deformities. Three RCTs demonstrated better functional outcomes of medial pivoting designs when compared to the conventional TKA. Only one RCT showed worse results in the medial pivoting group, whereas the remaining RCTs demonstrated non-significant differences between groups. However, several limitations and biases affect this review and further high-quality studies are needed to clarify the role of medial pivoting implants in TKA.

## Supplementary Information

Below is the link to the electronic supplementary material.

Supplementary file1 (DOCX 20 kb)
