# Supplementary material for: Clinical radiographic outcomes and survivorship of medial pivot design total knee arthroplasty: a systematic review of the literature
Source: Arch Orthop Trauma Surg. 2021 Oct 11;142(11):3437–48. doi: 10.1007/s00402-021-04210-6 (PMC9522696; doi:10.1007/s00402-021-04210-6)
Supplement: Supplementary file 1 — Supplementary file1 (DOCX 20 kb) [file 402_2021_4210_MOESM1_ESM.docx]

|  | ***Apriori GRADE*** | ***Risk of bias*** | ***Inconsistency of Results*** | ***Indirectness of evidence*** | ***Imprecision*** | ***Reporting Bias*** | ***Final GRADE*** |
| --- | --- | --- | --- | --- | --- | --- | --- |
| *Mannan* | Low | **Y** | **N** | **N** | **Y** | **N** | Very Low |
| *Fan* | Low | **Y** | **N** | **N** | **Y** | **N** | Very Low |
| *Hossain* | High | **Y** | **N** | **Y** | **N** | **N** | Low |
| *Vecchini* | Low | **Y** | **N** | **N** | **N** | **N** | Very Low |
| *Ishida* | High | **N** | **N** | **Y** | **N** | **N** | Moderate |
| *Brinkman* | Low | **Y** | **N** | **N** | **Y** | **N** | Very Low |
| *Seok Youm* | Low | **Y** | **N** | **N** | **N** | **N** | Very Low |
| *Chinzei* | Low | **Y** | **N** | **Y** | **N** | **N** | Very Low |
| *Schmidt* | Low | **Y** |  | **N** | **N** | **N** | Very Low |
| *Bae* | Low | **Y** | **N** | **N** | **N** | **N** | Very Low |
| *Katchky* | Low | **Y** | **N** | **N** | **N** | **N** | Very Low |
| *Nakamura* | Low | **Y** | **N** | **N** | **N** | **N** | Very Low |
| *Choi* | Low | **Y** | **N** | **N** | **N** | **N** | Very Low |
| *Karachalios* | Low | **Y** | **N** | **Y** | **N** | **N** | Very Low |
| *Macheras* | Low | **Y** | **N** | **N** | **N** | **N** | Very Low |
| *Dehl* | Low | **Y** | **N** | **N** | **N** | **N** | Very Low |
| *Kim* | High | **Y** | **N** | **N** | **Y** | **N** | Low |
| *Benjamin* | High | **Y** | **N** | **N** | **N** | **N** | Moderate |
| *Nakamura* | Low | **N** | **N** | **Y** | **N** | **N** | Very Low |
| *Samy* | Low | **Y** | **N** | **N** | **N** | **N** | Very Low |
| *Kohei* | High | **Y** | **N** | **N** | **Y** | **N** | Low |
| *Sabatini* | Low | **N** | **N** | **N** | **Y** | **N** | Very Low |
| *Karachalios* | Low | **Y** | **N** | **N** | **Y** | **N** | Very Low |
| *Cacciola* | Low | **Y** | **N** | **N** | **N** | **N** | Very Low |
| *Indelli* | High | **N** | **N** | **N** | **N** | **N** | High |
| *Gill* | High | **Y** | **N** | **N** | **Y** | **N** | Low |
| *French* | High | **Y** | **N** | **N** | **N** | **N** | Moderate |
| *Yuan* | Low | **N** | **N** | **N** | **N** | **N** | Low |
| *Indelli* | High | **N** | **N** | **Y** | **N** | **N** | Very low |
| *Lee* | High | **Y** | **N** | **Y** | **Y** | **N** | Very Low |
| *Jones* | Low | **Y** | **N** | **Y** | **Y** | **N** | Very Low |
| *Risitano* | Low | **Y** | **N** | **N** | **Y** | **N** | Very Low |
| *Edelstein* | High | **Y** | **N** | **N** | **Y** | **N** | Low |
| *Jeremic* | Low | **Y** | **N** | **N** | **Y** | **N** | Very Low |

GRADE Quality Assessment of included studies. N=No, Y= Yes
